# SARS-Coronavirus Open Reading Frame-3a drives multimodal necrotic cell death

**DOI:** 10.1038/s41419-018-0917-y

**Published:** 2018-09-05

**Authors:** Yuan Yue, Neel R. Nabar, Chong-Shan Shi, Olena Kamenyeva, Xun Xiao, Il-Young Hwang, Min Wang, John H. Kehrl

**Affiliations:** 1 State Key Laboratory of Oral Diseases & National Clinical Research Center for Oral Diseases, Department of Prosthodontics, West China Hospital of Stomatology, Sichuan University, Chengdu, 610041 China; 2 B Cell Molecular Immunology Section, Laboratory of Immunoregulation, National Institute of Allergy and Infectious Diseases, National Institutes of Health, Bethesda, MD 20892 USA; 3 Department of Molecular, Tumor, and Cell Biology, Karolinska Institutet, Stockholm, Sweden 17165; 4 Biological Imaging Section, Research Technologies Branch, National Institute of Allergy and Infectious Diseases, National Institutes of Health, Bethesda, MD 20892 USA

## Abstract

The molecular mechanisms underlying the severe lung pathology that occurs during SARS-CoV infections remain incompletely understood. The largest of the SARS-CoV accessory protein open reading frames (SARS 3a) oligomerizes, dynamically inserting into late endosomal, lysosomal, and trans-Golgi-network membranes. While previously implicated in a non-inflammatory apoptotic cell death pathway, here we extend the range of SARS 3a pathophysiologic targets by examining its effects on necrotic cell death pathways. We show that SARS 3a interacts with Receptor Interacting Protein 3 (Rip3), which augments the oligomerization of SARS 3a helping drive necrotic cell death. In addition, by inserting into lysosomal membranes SARS 3a triggers lysosomal damage and dysfunction. Consequently, Transcription Factor EB (TFEB) translocates to the nucleus increasing the transcription of autophagy- and lysosome-related genes. Finally, SARS 3a activates caspase-1 either directly or via an enhanced potassium efflux, which triggers NLRP3 inflammasome assembly. In summary, Rip3-mediated oligomerization of SARS 3a causes necrotic cell death, lysosomal damage, and caspase-1 activation—all likely contributing to the clinical manifestations of SARS-CoV infection.

## Introduction

Severe acute respiratory syndrome (SARS) is caused by a coronavirus (SARS-CoV) that at its peak affected more than 8000 people with a 10% mortality rate^1^. The recent emergence of a SARS-like CoV called Middle East Respiratory Syndrome coronavirus has underscored the need to understand the mechanisms behind CoV pathogenicity^2^. SARS presents with flu-like symptoms that can progress to respiratory failure secondary to immunopathologic injury^3,4^. Pathologic examination of lung tissue from fatal cases shows diffuse alveolar damage, significant monocyte–macrophage infiltration, and elevated serum cytokines^3,5,6^. A study in mouse models highlighted the importance of inflammatory monocyte-macrophages (IMMs) in SARS pathogenesis^7^, as high initial viral titer along with delayed type I interferon induction results in the recruitment and aberrant activation of IMMs. Deletion of the interferon receptor or IMMs rescued pathologic elevation of these cytokines post-infection and prevented lethal infection in mouse models, underscoring that patient death is likely due to a combination of an aberrant innate immune response and direct cytopathic effects of the virus^7^.

While the contributions of IMMs to disease pathogenesis is now understood, the molecular mechanisms behind their aberrant inflammatory state is not. The SARS-CoV genome encodes eight accessory proteins designated open reading frame (ORF)-3a, 3b, 6, 7a, 7b, 8a, 8b, and 9b^8^. Several ORF functions have been identified: ORF-7a activates NF-κB;^9^ ORF3b upregulates the expression of several cytokines and chemokines;^10,11^ ORF-6 reduces IFN production;^12^ ORF-8a triggers cellular apoptosis;^13^ and ORF-8b reduces viral replication^14^. ORF-9b targets the MAVS signalosome to trigger the degradation of MAVS, TRAF3, and TRAF6, severely limiting the host cell IFN response^15^. However, in apparent contradiction with the severe inflammatory phenotype important in SARS pathogenesis, the SARS-CoV accessory proteins thus far have primarily been implicated in apoptotic (non-inflammatory) cell death. Cells undergoing apoptosis show morphological apoptotic hallmarks of cell shrinkage and nuclear fragmentation^16^, which limits the inflammatory response by neatly containing dying cells for clearance by macrophages^17,18^. Necrotic cell death is inflammatory in nature due to the release of intracellular contents and is morphologically characterized by a gain in cell volume, organelle swelling, and plasma membrane rupture^18,19^.

Recent advances have discovered multiple pathways of programmed necrosis, including necroptosis and pyroptosis. Necroptosis is a caspase-independent form of programmed necrosis mediated by the Rip1–Rip3–MLKL signaling axis. Activated Rip3 phosphorylates MLKL, inducing its oligomerization, membrane insertion, and pore formation^20^. Pyroptosis is another form of inflammatory cell death following inflammasome activation; it allows the release of proinflammatory damage associated molecular patterns^21^. Inflammasome activation occurs when pathogenic molecules or cell stress activates the inflammasome sensor proteins, which then form a multimeric complex that directly activates caspase-1, allowing the cleavage of pro-IL-1β to its mature form^22^. Activated caspase-1 also cleaves the effector molecule Gasdermin D, which oligomerizes and inserts into the plasma membrane to form pores^23^. Importantly, both forms of inflammatory cell death share a similar final effector step, namely the insertion of an oligomerized protein with channel functionality into the plasma membrane.

The SARS-CoV ORF-3a protein (SARS 3a), at 274 amino-acids, is the largest SARS-CoV accessory protein^8^. The N-terminus of SARS 3a contains three transmembrane segments, and disulfide bond formation at cysteine-133 mediates its oligomerization and ion channel functionality^24^. Deletion studies from live virus indicate that ORF-3a is critical for SARS-CoV-infected cell death, and in vivo murine studies show that deletion of ORF-3a rescues mice from SARS-CoV-induced death^25,26^. Importantly, overexpression of GFP-tagged SARS 3a accurately recapitulates the cell death phenotype^25^. As SARS 3a shares membrane insertion characteristics and channel functionality with necrotic effector molecules, we investigated the downstream consequences of SARS 3a membrane insertion.

## Results

### SARS 3a-associated necrotic cell death is Rip3 dependent

**Fig 1:**
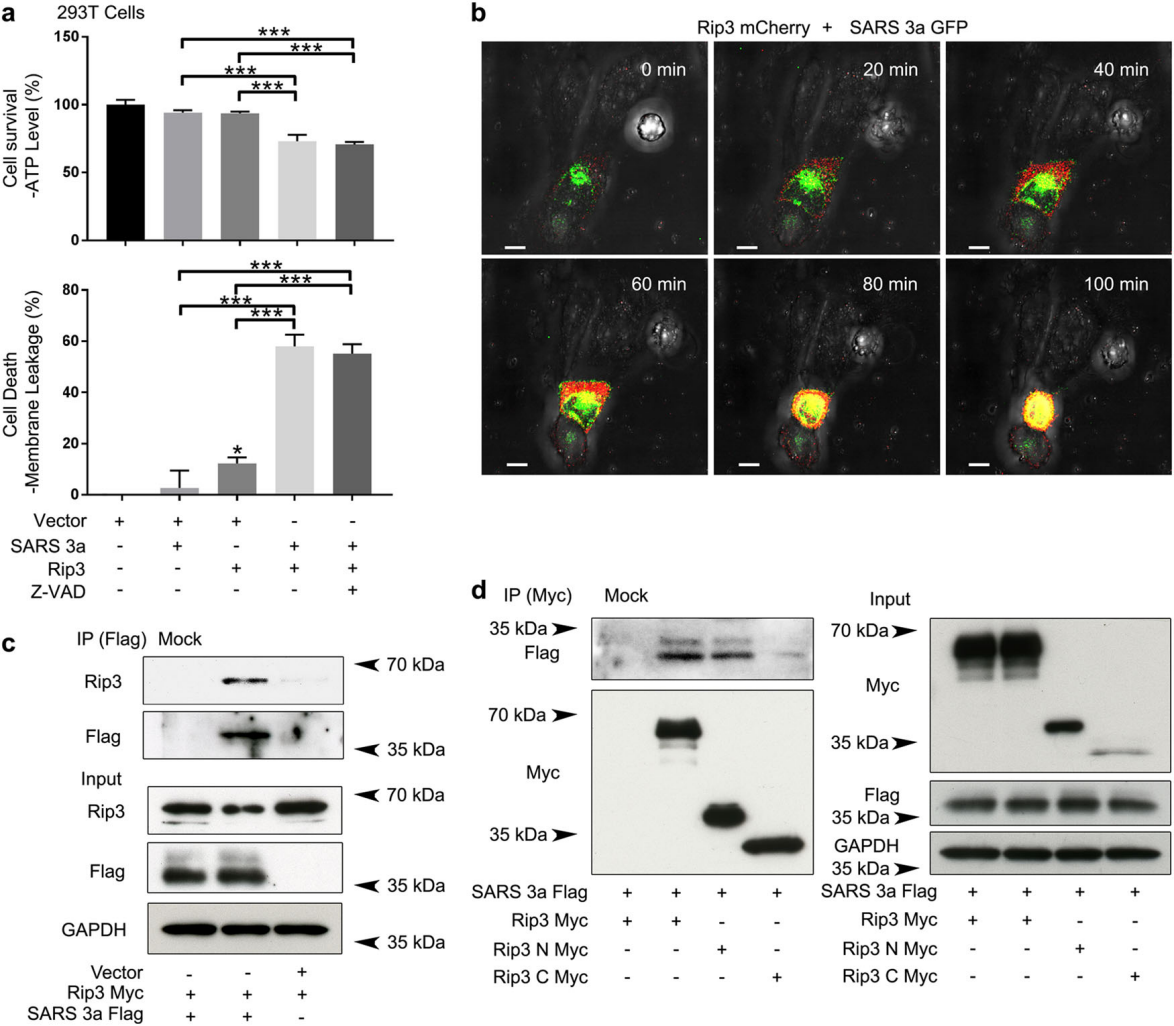
SARS 3a drives Rip3-dependent necrotic cell death. **a** Cell death assays (ATP level and membrane leakage assays) in HEK 293T cells after co-transfection of SARS 3a and Rip3, with and without caspase inhibitor. **b** Confocal time lapse showing cell death after transfection of SARS 3a-GFP in the presence of Rip3-mCherry in HeLa cells (Scale bar, 10 μm). **c** Immunoprecipitation analysis of the interaction between SARS 3a and Rip3 in HEK 293T cells. **d** Immunoblot of Myc immunoprecipitate from HEK 293T cells expressing full-length or truncated Rip3-Myc and SARS 3a-flag. Myc and flag expression levels were verified by immunoblotting cell lysates from the same cells. The western data are representative of two or three independent experiments. Cell death data are the average and SEM of *n* = 3 independent experiments in triplicate (*p < 0.05; **p < 0.001, ***p < 0.0001, ordinary one-way ANOVA with post hoc Tukey’s HSD)

Rip3 mediates necroptosis by phosphorylating MLKL, resulting in its oligomerization and membrane insertion. SARS 3a has three transmembrane segments, allowing it to form a potassium-sensitive channel when oligomerized^24^. Based on the shared ability of MLKL and SARS 3a to oligomerize and insert into membranes, we addressed whether Rip3 could target SARS 3a to drive necrotic cell death. To assay necrotic cell death, we concomitantly monitored cellular ATP (which is depleted during death) and the release of cytosolic proteases (which increases with necrosis)^27^. To parse out contributions from components of the necroptotic pathway, we used 293T and HeLa cells in parallel, as 293T cells do not express endogenous Rip3 or MLKL, while HeLa cells express MLKL but not Rip3^28^. Overexpression of SARS 3a in 293T or HeLa cells alone did not cause significant ATP depletion or the release of cellular proteases. Similarly, expression of Rip3 in 293T cells expectedly did not cause necrotic cell death due to the lack of necroptosis effector MLKL. However, co-expression of SARS 3a and Rip3 caused substantial necrotic death that was unaffected by the addition of a pan-caspase inhibitor, suggesting Rip3 helps SARS 3a cause necrotic death in a caspase-independent manner (Fig. 1a, Supplementary Figure 1). To directly observe cell death, we transfected Rip3-mCherry into HeLa cells expressing SARS 3a-GFP and recorded the cell death process by time-lapse confocal microscopy. The images show that expression of Rip3 drives cell death in the presence of SARS 3a. As a control, a neighboring cell which did not express SARS 3a and Rip3 was captured and clearly survives the duration of imaging (Fig. 1b, video 1). The above functional results encouraged us to determine if SARS 3a and Rip3 interact by immunoprecipitation. After expression of SARS 3a-flag and Rip3-Myc in 293T cells, immunoprecipitated SARS 3a-flag pulls down Rip3-Myc, whereas SARS 3a and Rip3-Myc cannot be detected from immunoprecipitation using mock antibody beads (Fig. 1c). Mapping of the interaction between Rip3 and SARS 3a showed that the kinase domain of Rip3 (1–326) interacted with SARS 3a, but that the RHIM motif containing C-terminus (327–518) interacted very weakly (Fig. 1d).

### Rip3 induced oligomerization of SARS 3a drives necrotic cell death

**Fig. 2:**
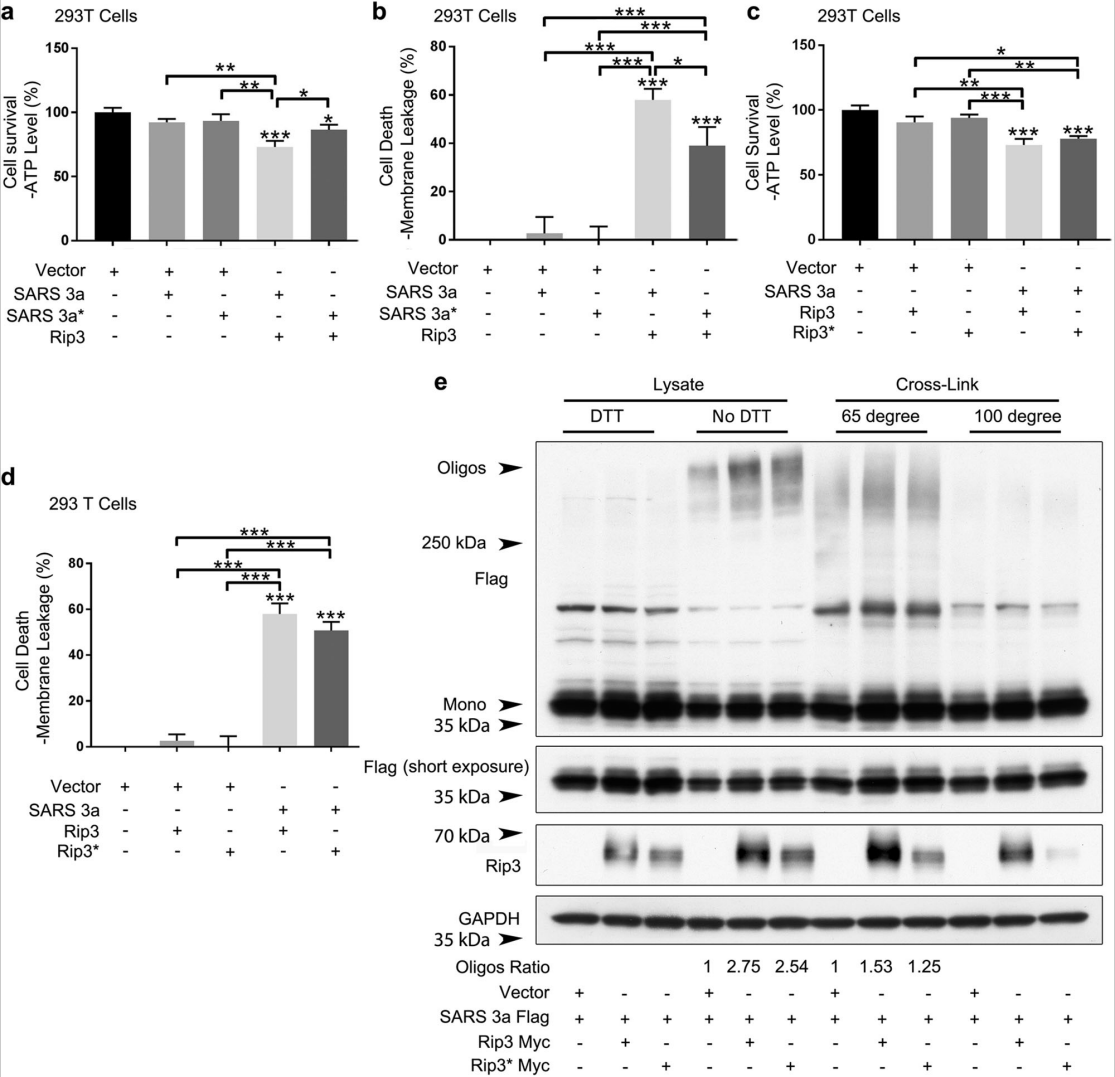
SARS 3a induced cell death is SARS 3a oligomerization dependent and Rip3 kinase independent. **a**, **b** Cell death assays (ATP level and membrane leakage assays) in HEK 293T cells following co-transfection of Rip3 and either SARS 3a WT or SARS 3a C133A (SARS 3a*). **c**, **d** Cell death assays in HEK 293T cells after co-transfection of SARS 3a and either Rip3 WT or Rip3 kinase dead (Rip3*). **e** Western blot analysis of cell lysates or cross-linked samples from HEK 293T cells to determine oligomerization of SARS 3a in the presence of Rip3. The western blot data are representative of two or three independent experiments. Cell death data are the average and SEM of *n* = 3 independent experiments in triplicate (*p < 0.05; **p < 0.001, ***p < 0.0001, ordinary one-way ANOVA with post hoc Tukey’s HSD)

To address whether oligomerization plays a role in SARS 3a-driven necrotic cell death, we transfected an oligomerization-deficient SARS 3a-flag C133A mutant^24^ into 293T or Hela cells and monitored necrotic cell death. While expression of SARS 3a C133A alone showed similar behavior to WT SARS 3a (minimal necrotic death), the SARS 3a C133A mutant induced significantly less necrotic death than the WT upon co-transfection with Rip3 (Fig. 2a, b; Supplementary Figure 2a, b), indicating that the oligomerization of SARS 3a is important for Rip3/SARS 3a-driven necrotic cell death. To determine whether Rip3 kinase activity is required to drive SARS 3a-mediated death, the Rip3 K50A (kinase dead) mutant was used in the above assays. The results showed that the Rip3 kinase dead form induced necrotic cell death to a similar extent as WT Rip3 when expressed with SARS 3a (Fig. 2c, d; Supplementary Figure 2c, d).

We then checked whether Rip3 enhances the oligomerization of SARS 3a. Using immunoblotting to detect SARS 3a oligomerization, we found that expression of both wild-type Rip3 and its kinase dead form increased SARS 3a oligomerization (2.75× and 2.54×, respectively); the addition of DTT completely erases the oligomerization, indicating it is disulfide bond dependent as seen in previous studies^24^. As a parallel experiment, the oligomerization assay was done in cells treated with a protein crosslinker. When samples were heated at 65 °C, SARS 3a oligomerization increases 1.53× and 1.25× after transfection of WT and kinase dead Rip3, while heating samples to 100 °C abrogated detectable oligomerization (Fig. 2e). We note that the oligomerization-deficient mutant causes some cell death (Fig. 2a, b), due to either residual oligomerization activity of the mutant or cell death by the monomer. Nevertheless, our results demonstrate that Rip3-driven oligomerization of SARS 3a plays a critical role in driving necrotic cell death, but that Rip3 kinase activity is dispensible.

### MLKL is dispensable for necrotic cell death driven by Rip3 and SARS 3a

**Fig. 3:**
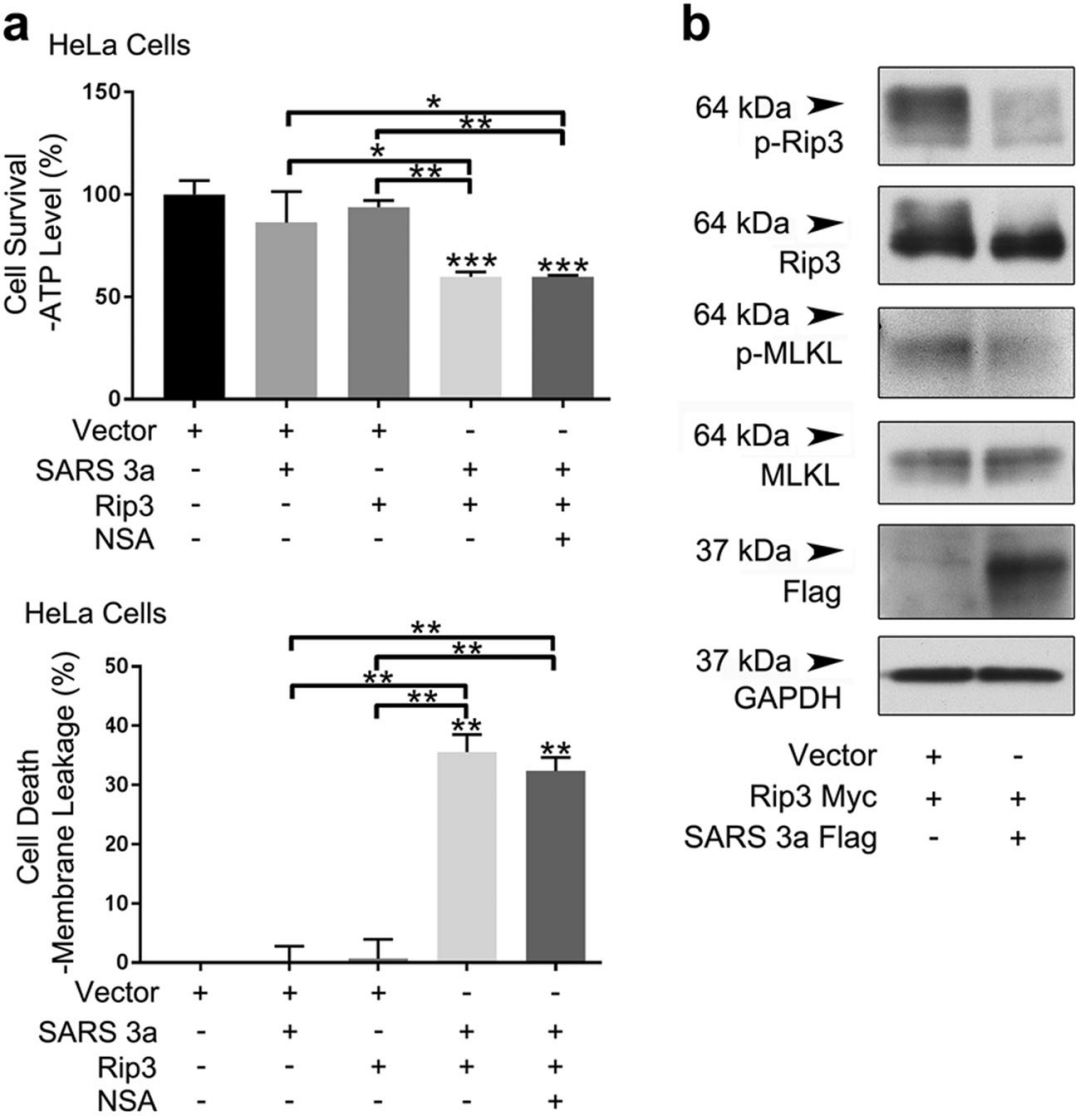
SARS 3a induced cell death is MLKL independent. **a** Cell death assays (ATP level and membrane leakage assays) in HeLA cells co-transfected with SARS 3a and Rip3 with or without an MLKL inhibitor (NSA). **b** Immunoblot of lysates from HeLa cells expressing SARS 3a-flag and Rip3-Myc. Western data are representative of two or three independent experiments. Cell death data are the average and SEM of *n* = 3 independent experiments in triplicate (*p < 0.05; **p < 0.001, ***p < 0.0001, ordinary one-way ANOVA with post hoc Tukey’s HSD)

Given that HeLa cells express endogenous MLKL, and co-expression of Rip3 and SARS 3a caused necrotic cell death, we addressed whether SARS 3a/Rip3-mediated cell death was MLKL dependent. Addition of an MLKL inhibitor did not reduce necrotic cell death driven by the co-expression of Rip3 and SARS 3a (Fig. 3a). This is consistent with our earlier data showing cell death in 293T cells following expression of Rip3 and SARS 3a (Fig. 1a). Furthermore, expression of SARS 3a reduced phosphorylation of both transfected Rip3 and endogenous MLKL (Fig. 3b). The assayed phosphorylation sites of Rip3 and MLKL are indicators of necroptotic signaling^27,29^. Although SARS 3a interacts with Rip3 and Rip3 enhances oligomerization of SARS 3a causing necrotic cell death, this appears to be independent from, and may even partially inhibit Rip3-MLKL necroptotic signaling. Thus, given that SARS 3a can insert into membranes and act as an ion channel^24^, our data suggest that SARS 3a may hijack host cell necroptotic machinery and directly act as a necrotic cell death effector regulated by Rip3.

### SARS 3a causes cell death in a human lung cell line after induction of necroptotic elements

**Fig. 4:**
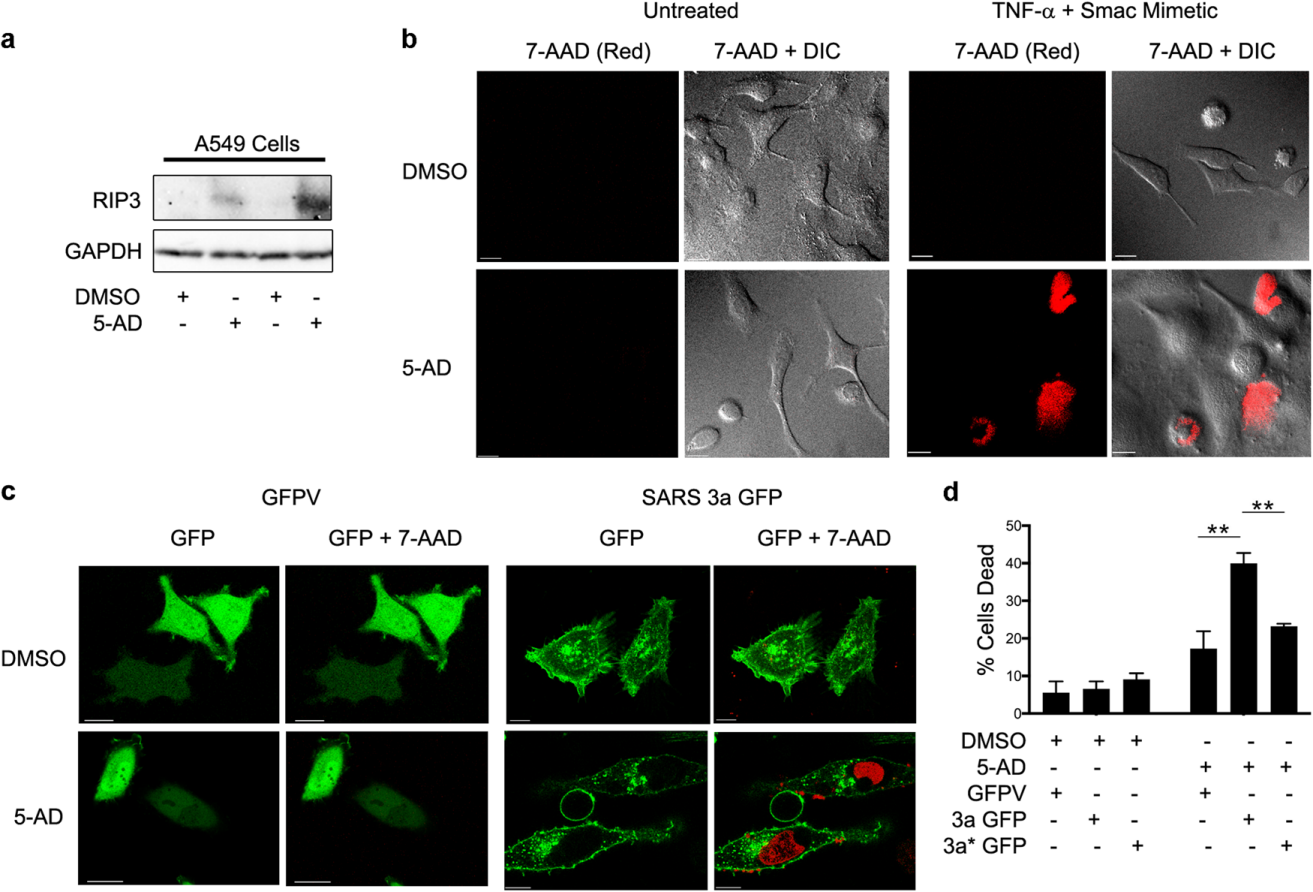
SARS 3a induces cell death in a human lung cell line with an intact necroptotic pathway. **a** Immunoblots for the indicated proteins after treatment of A549 cells with 5-AD (2 μm) or DMSO for 4 days. **b** Confocal microscopy to evaluate cell death by 7-AAD staining (Red) in DMSO- or 5-AD (2 μM)-treated A459 cells after necroptotosis-inducing treatment (TNF-α (25 ng/mL), Z-VAD-FMK (20 μM), BV6 (2 nM)) overnight (×63 electronic zoom, scale bar 15 μm). **c** Confocal microscopy of 5-AD (2 μM) or DMSO-treated A459 cells transiently expressing GFP-vector (GFPV) or SARS 3a-GFP stained with 7-AAD (Red) (×63 electronic zoom, scale bar 10 μm). **d** Quantification of cell viability in GFP-positive cells after transfection of GFP-vector (GFPV), SARS 3a-GFP (3a-GFP), or SARS 3a C133A-GFP (3a* GFP). Cell death data are the average and SEM of *n* = 3 experiments (*p < 0.05; **p < 0.001, ***p < 0.0001, ordinary one-way ANOVA with post hoc Tukey’s HSD)

We next investigated whether SARS 3a could cause cell death in a lung cell line with an intact endogenous necroptotic pathway. Rip3 is suppressed in most cancer cell lines by DNA methylation, and treatment with hypomethylating agents such as 5-Aza-2′-deoxycytidine (5-AD) induces Rip3 expression and sensitizes these cells to necroptosis inducing stimuli^30^. Previous reports have shown that the human alveolar epithelial cell line A549 is resistant to the traditional necroptotic stimulus (TNF-α + caspase inhibitor Z-VAD-FMK + Smac-mimetic BV6), and that treatment with 5-AD induces endogenous Rip3 expression^30^. To validate this system, we first treated A459 cells with 5-AD or DMSO, followed by immunoblotting for Rip3. As expected, Rip3 was undetectable in the DMSO-treated cells, but clearly induced by 5-AD (Fig. 4a). Consistently, DMSO-treated cells showed very little cell death after TNF-α + Z-VAD-FMK + Smac-mimetic treatment as assayed by 7-aminoactinomycin D (7-AAD, red color) staining, while 5-AD treated cells under the same conditions showed significant cell death (~50%) (Fig. 4b). We next transfected DMSO- or 5-AD-treated A549 cells with GFP-vector, SARS 3a-GFP, or SARS 3a C133A-GFP and imaged them live after 7-AAD staining. In the DMSO-treated cells, transfection of SARS 3a-GFP or its mutant did not induce cell death to a greater extent than the control. However, transfection of SARS 3a-GFP induced significant cell death in 5-AD-treated cells, while cell death was reduced almost to basal levels in SARS 3a C133A-GFP-transfected cells (Fig. 4c, d). Taken together, these data suggest that SARS 3a does not induce cell death in the absence of Rip3, but induces significant oligomerization-dependent death in the presence of endogenous Rip3.

### SARS 3a causes lysosome damage

**Fig. 5:**
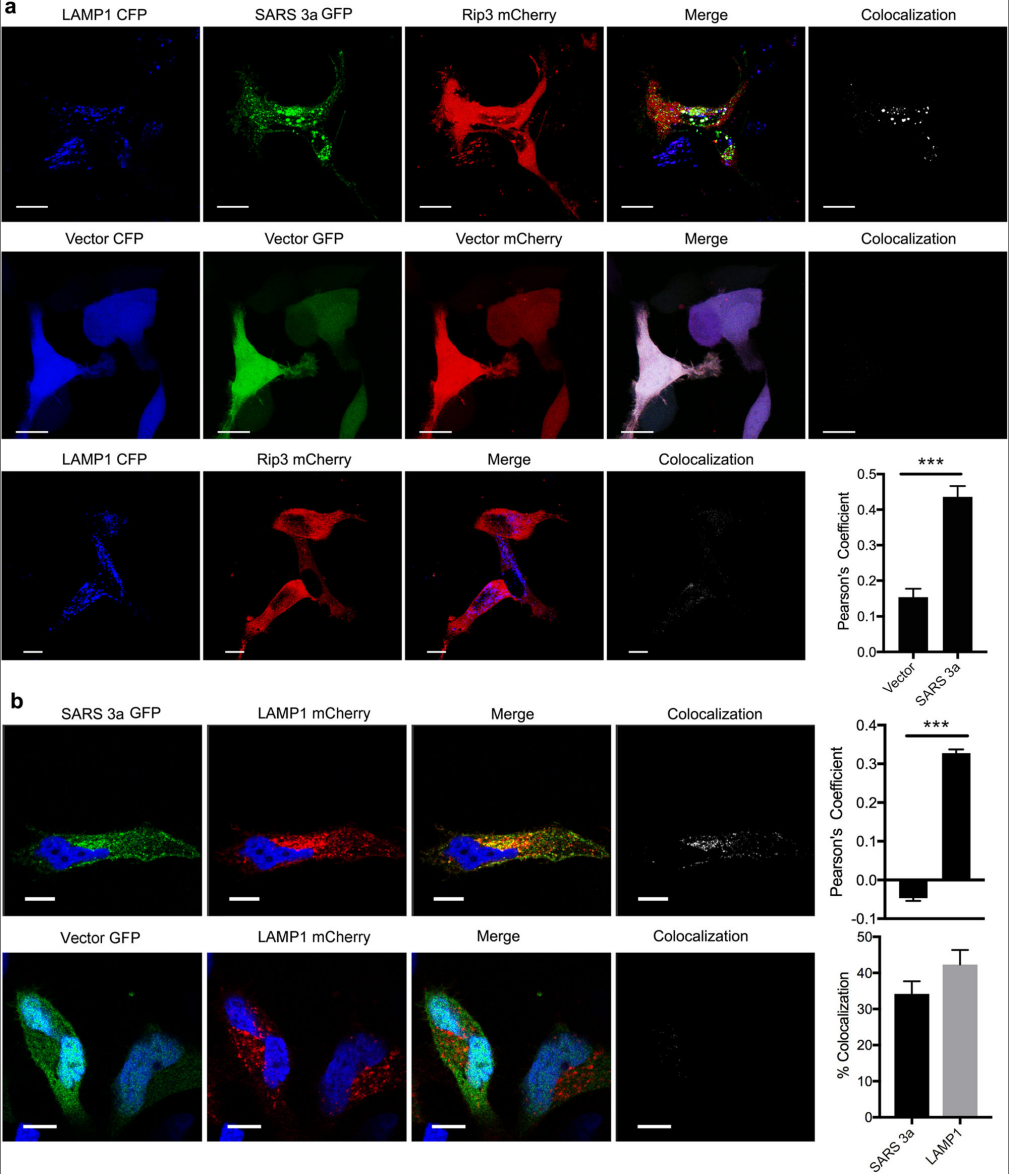
SARS 3a and Rip3 co-localize to the lysosome. Confocal imaging analysis from confocal microscopy of (**a**) HeLa cells expressing LAMP1-CFP, SARS 3a-GFP, and Rip3-mCherry 12 h after transfection. Pearson's correlation coefficient (PCC) between LAMP1-CFP and Rip3-mCherry with and without SARS 3a-GFP is shown on the right (*n* = 15 cells). **b** HeLa cells expressing LAMP1-mCherry and SARS 3a-GFP to determine subcellular localization. Pearson's correlation coefficient (PCC) and percent colocalization between LAMP1-mCherry and SARS 3a-GFP are shown on the right (*n* = 15 cells). ×100 electronic zoom, scale bar 10 μm (***p < 0.0001, Unpaired Student’s *T*-test)

**Fig. 6:**
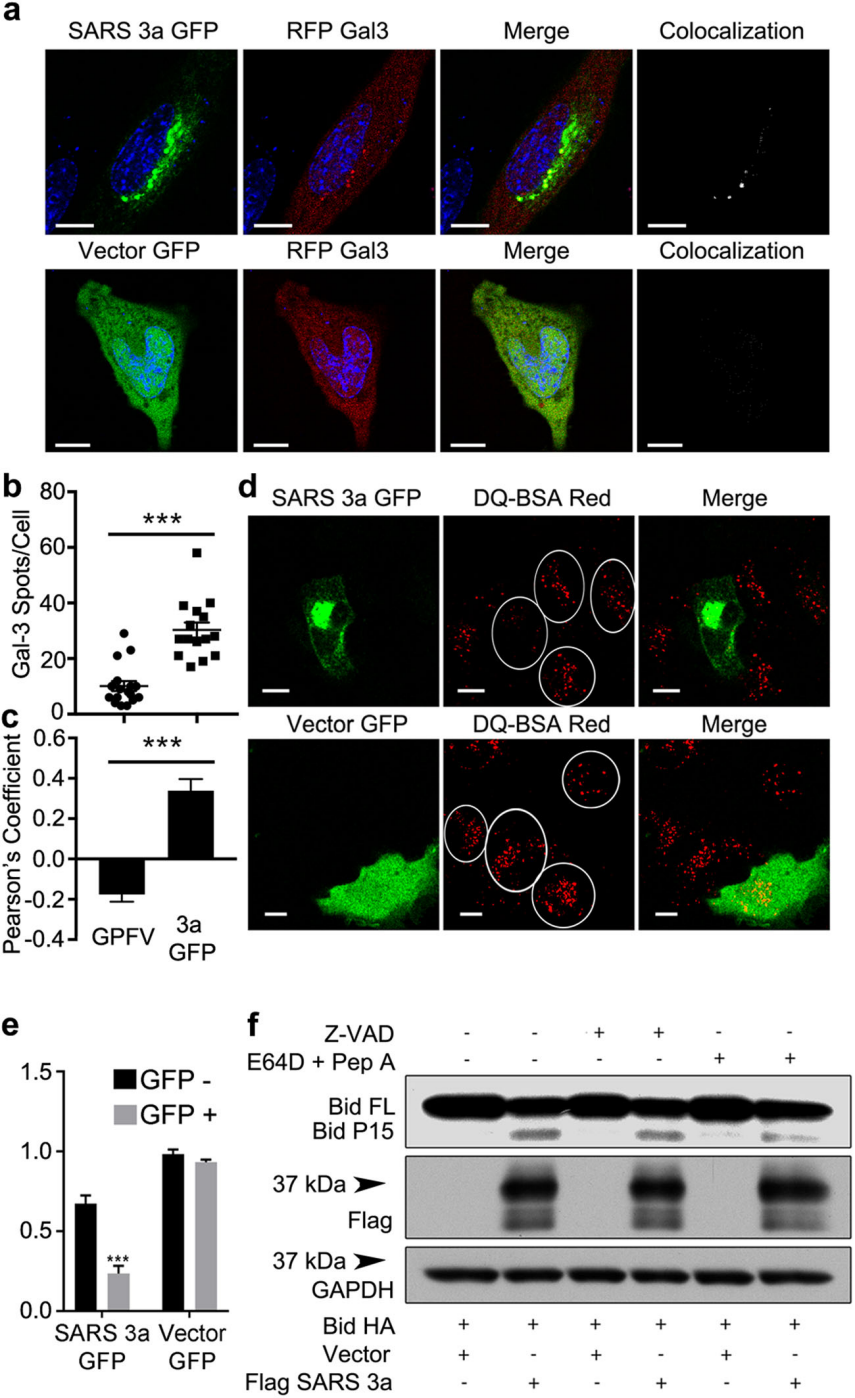
SARS 3a causes lysosome damage. Confocal imaging analysis of (**a**) HeLa cells co-transfected with Galectin3-mCherry and SARS 3a-GFP, to assay lysosomal damage. **b** Quantification of Galectin3-mCherry spots/cell from GFP-vector (GPFV) or SARS 3a-GFP (3a GFP) expressing cells (*n* = 15 cells). **c** Quantification of colocalization (PCC) between Galectin3-mCherry and either GFP-vector or SARS 3a-GFP (*n* = 15 cells). **d** HeLa cells transfected with SARS 3a-GFP and incubated with DQ-BSA red to assay lysosomal degradation capacity. ×100 electronic zoom, scale bar 10 μm. **e** Quantification of multiple images from **d**, data shown as normalized intensity. **f** Immunoblot of lysates from 293T cells expressing SARS 3a-flag and HA-bid with or without Z-VAD or E64D and pep A. The western data are representative of two or three independent experiments (***p < 0.0001, unpaired Student’s *T-*test)

Next, we investigated whether SARS 3a affects lysosome function by inserting into lysosomal membranes. To do so we co-transfected SARS 3a-GFP, Rip3-mCherry, and LAMP1-CFP (a lysosome membrane marker) into HeLa cells. Confocal imaging showed that co-expressed SARS 3a and Rip3 co-localize with LAMP1, but that Rip3 only minimally localizes to the lysosome without SARS 3a (Fig. 5a). Consistently, quantification of colocalization between Rip3 and LAMP1 by Pearson’s Correlation Coefficient (PCC) showed an increase from 0.153 basally to 0.435 after co-transfection with SARS 3a (Fig. 5a), indicating that SARS 3a likely targets Rip3 to lysosomes, SARS 3a alone localized with LAMP1 (Fig. 5b) (PCC = 0.330, 34% SARS 3a-GFP and 42% LAMP1-mCherry colocalizing). To test whether SARS 3a causes lysosome damage, we monitored Galectin 3, which forms puncta on the lysosome after lysosomal membrane permeabilization^31^. Co-transfection of RFP-Galectin 3 and SARS 3a-GFP showed significantly more Galectin-3 puncta per cell than the control GFP-vector (Fig. 6a, b). We note SARS 3a and Galectin 3 colocalized (PCC = 0.339), indicating that the lysosome damage is SARS 3a specific (Fig. 6c). To validate that SARS 3a causes lysosomal damage, we assayed the lysosomal degradation capacity of SARS 3a-transfected cells using DQ-BSA red dye, which fluoresces after activation by lysosomal proteases. Cells expressing SARS 3a-GFP had dramatically decreased DQ-BSA red stain compared with the GFP-vector control (Fig. 6d), and intensity analysis from several images confirmed that SARS 3a-GFP-positive cells showed a significant decrease of the DQ-BSA stain (Fig. 6e). Finally, to test whether lysosomal cathepsins were released into the cytoplasm from SARS 3a-damaged lysosomes, we transfected a cytoplasmic cathepsin substrate Bid and assayed Bid cleavage by immunoblot. Expression of SARS 3a induced cleavage of Bid, which was reversed by the addition of the cathepsin inhibitors E64D and pepstatin A (Fig. 6f). These data indicate that SARS 3a causes lysosomal membrane permeablization and the release of cathepsins from lysosomes, resulting in impaired lysosomal degradation capacity.

### SARS 3a induces TFEB activation

**Fig. 7:**
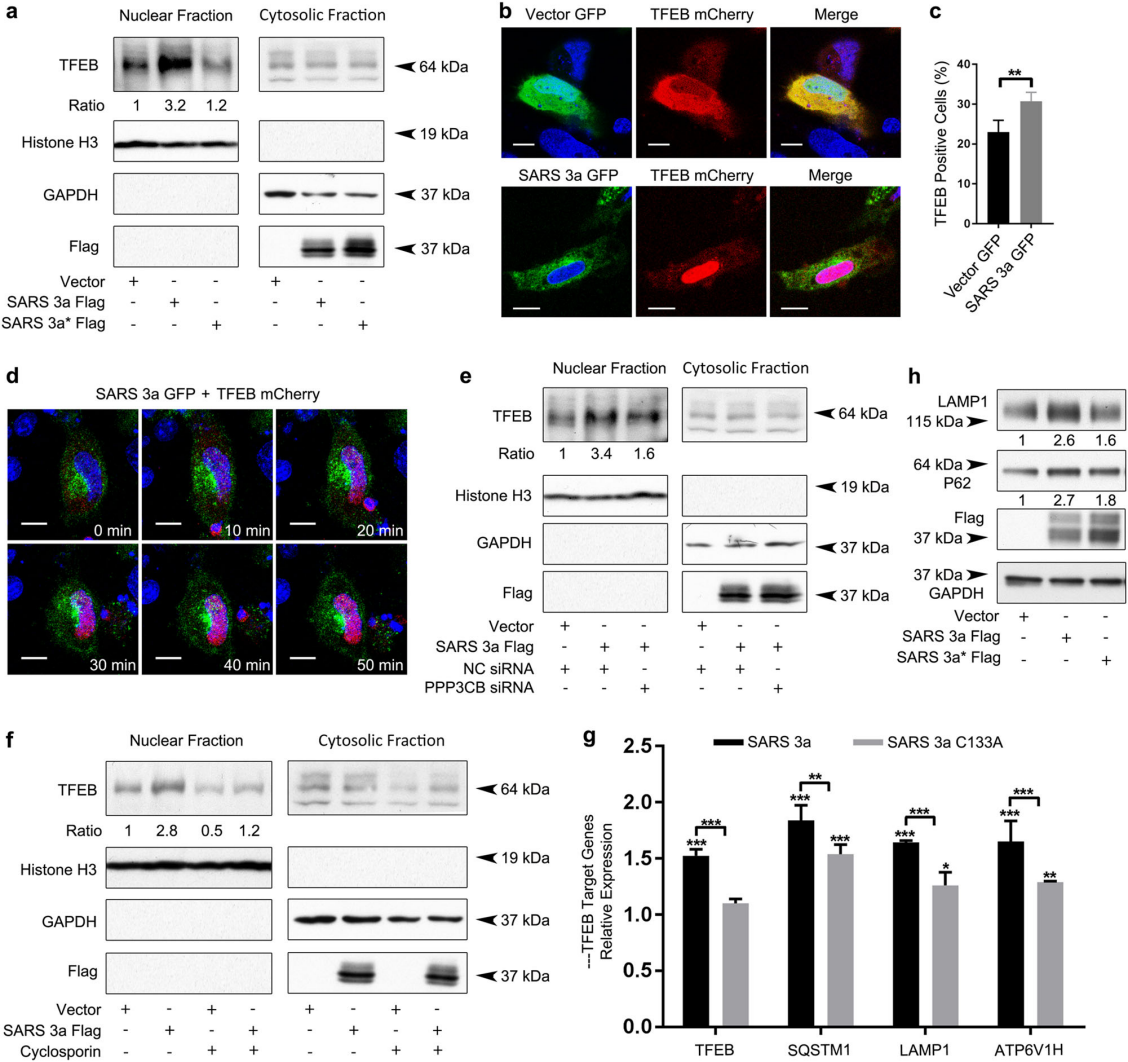
SARS 3a drives TFEB translocation to nucleus via calcineurin. **a** Immunoblots showing endogenous TFEB after nuclear fractionation of 293T cells transfected with SARS 3a-flag or SARS 3a C133A-flag (SARS 3a* flag). Histone H3 and GAPDH are used as fractionation and loading controls. **b** Subcellular localization analysis by confocal microscopy of HeLa cells expressing TFEB-mCherry with SARS 3a-GFP or a GFP-vector. **c** Quantification of immunofluorescence data in **b.** **d** Live time-lapse confocal microscopy of SARS 3a-GFP driving TFEB-mCherry nuclear translocation, after co-expression of SARS 3a-GFP and TFEB-mCherry. **e** Immunoblot showing endogenous TFEB after nuclear fractionation of 293T cell lysates after transfection with SARS 3a-flag and either PPP3CB or control siRNA. **f** Immunoblot showing endogenous TFEB after nuclear fractionation of 293T cell lysates after transfection with SARS 3a-flag and treatment with cyclosporine. **g** qRT-PCR expression analysis of TFEB target genes after SARS 3a or SARS 3a C133A overexpression. Bars show the mRNA level fold change normalized to GAPDH and relative to the vector control. **h** Immunoblot analysis of TFEB target proteins expression level after transfection with SARS 3a-flag or SARS 3a C133A-flag (SARS 3a* flag). All western data are representative of two or three independent experiments. Western bands are quantified and normalized to the loading control then presented relative to the control lane. Confocal imaging done at ×100 electronic zoom, scale bar 10 μm. qRT-PCR data are the average and SEM of *n* = 3 independent experiments in triplicate (*p < 0.05; **p < 0.001, ***p < 0.0001, ordinary one-way ANOVA with post hoc Tukey’s HSD)

A variety of cellular stresses, including lysosomal dysfunction, cause activation of TFEB^32–34^. As SARS 3a causes lysosomal damage, we looked to see if SARS 3a induced TFEB nuclear translocation by separating the cytosolic and nuclear fraction from 293T cells and detecting endogenous TFEB. SARS 3a strongly induced TFEB nuclear translocation (3.2-fold greater than the control), while the oligomerization-deficient mutant did not (Fig. 7a). Consistenly, confocal microscopy of cells expressing SARS 3a-GFP and TFEB-mCherry showed that SARS 3a-GFP induced TFEB-mCherry nuclear translocation, but that a control GFP-vector did not (Fig. 7b, c).

To dynamically observe TFEB translocation after SARS 3a expression, we transfected SARS 3a-GFP and TFEB-mCherry into HeLa cells and recorded images via confocal time-lapse microscopy. In the presence of SARS 3a, it took 30 min to translocate all detectable TFEB into the nucleus (Fig. 7d, video 2). Under normal conditions, TFEB is phosphorylated by mTORC1 and sequestered in the cytoplasm. The phosphatase activity of calcineurin (PPP3CB) is essential in dephosphorylating TFEB to promote its nuclear translocation^35^. To address whether PPP3CB mediates SARS 3a-induced TFEB nuclear translocation, we used cyclosporine (a calcineurin inhibitor) and PPP3CB siRNA and found that both reduced SARS 3a-induced TFEB nuclear translocation (Fig. 7e, f). We next checked whether SARS 3a mediated TFEB nuclear translocation-induced TFEB target gene expression. qRT-PCR of LAMP1 and p62 showed increased transcript levels compared to the vector, while the SARS 3a C133A mutant did not induce those genes (Fig. 7g). Similarly, immunoblot analysis of LAMP1 and p62 confirmed increase in protein levels after SARS 3a transfection, but less induction after expression of the SARS 3a C133A mutant (Fig. 7h). Collectively, SARS 3a oligomerization induces TFEB nuclear translocation, activation, and induction of TFEB target genes via PPP3CB.

### SARS 3a activates NLRP3 inflammasomes

**Fig. 8:**
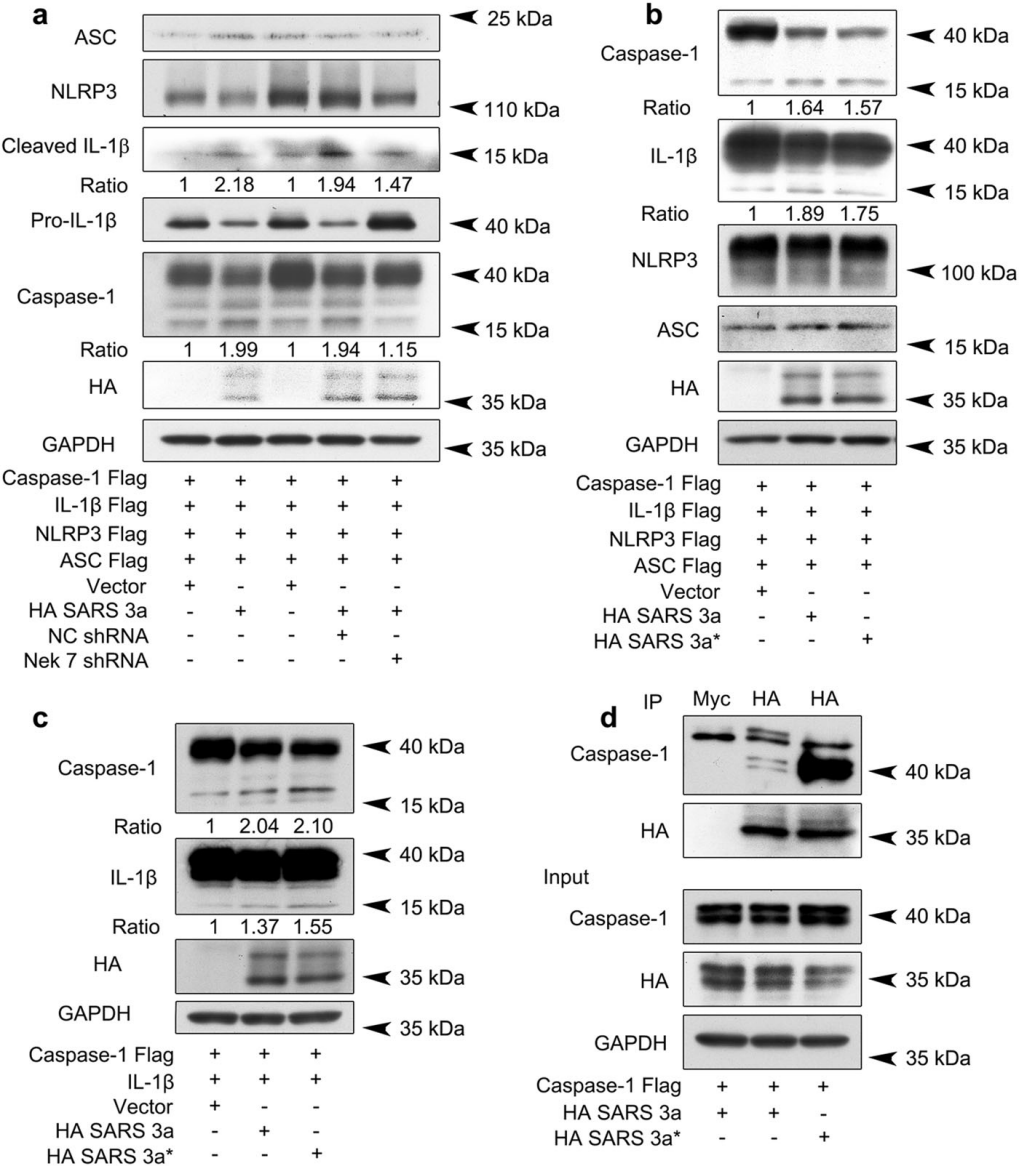
SARS 3a induces NLRP3 inflammasome activation by multiple mechanisms. **a** Immunoblot analysis of the pro- and cleaved forms of caspase-1 and IL-1β after reconstitution of inflammasome in HEK 293T cells transfected with SARS 3a with or without NEK7 shRNA. **b** Immunoblot analysis of the pro- and cleaved forms of caspase-1 and IL-1β after reconstitution of inflammasome and transfection with SARS 3a or SARS 3a C133A. **c** Immunoblot analysis of the pro- and cleaved forms of caspase-1 and IL-1β after co-transfection with caspase-1, IL-1β, and SARS 3a or SARS 3a C133A. **d** Immunoprecipitation analysis of interaction between SARS 3a or SARS 3a C133A and caspase-1. All western blot data are representative of two or three independent experiments

The NLRP3 inflammasome responds to a variety of pathogens and cell stress signals, and upon activation NLRP3 assembles with the adaptor protein ASC to form the active inflammasome that cleaves caspase-1. Potassium efflux is the most upstream shared signaling event across various NLRP3 activators, and NEK7 is an essential downstream kinase that mediates NLRP3 inflammasome assembly^36^. Given the ability of SARS 3a to act as a potassium channel^24^, we tested whether SARS 3a triggers the NLRP3 inflammasome. Using an NLRP3 inflammasome reconstitution system in 293T cells, expression of SARS 3a induced approximately twice as much caspase-1 activation and mature IL-1β formation than the control vector. To determine whether SARS 3a functioned upstream of NEK7 (which is directly downstream of potassium efflux), we knocked down NEK7 in the presence of SARS 3a. Compared to transfection of SARS 3a with control shRNA, NEK7 knockdown resulted in reduced cleaved caspase-1 and less mature IL-1β, confirming that SARS 3a acts upstream to NEK7 (Fig. 8a). We then transfected SARS 3a into Phorbol 12-myristate 13-acetate (PMA) differentiated Thp-1 macrophages, and observed the release of endogenous cleaved caspase-1 in culture supernatant (Supplementary Figure 3).

However, upon testing the SARS 3a C133A mutant we unexpectedly found that it activated the NLRP3 inflammasome to a similar extent as WT SARS 3a (Fig. 8b), indicating additional mechanisms exist by which SARS 3a activates NLRP3. We hypothesized that SARS 3a activates caspase-1 without NLRP3 and ASC. Consistenly, SARS 3a and its mutant produced more cleaved caspase-1 (2.04- and 2.10-fold) and IL-1β (1.37- and 1.55-fold) than basal even in the absence of NLRP3 and ASC (Fig. 8c). Based on these results, we checked if SARS 3a interacts directly with caspase-1; our data show that while wild-type SARS 3a interacts with caspase-1, the SARS 3a oligomerization-deficient mutant pulls down caspase-1 to a much greater degree (Fig. 8d). This result suggests that monomeric SARS 3a may have a higher affinity for caspase-1, and that SARS 3a activates the NLRP3 inflammasome both via potassium efflux mediated by SARS 3a oligomers and via direct caspase-1 targeting by SARS 3a monomers.

## Discussion

The role of the necroptotic pathway in antiviral immunity remains incompletely understood. Some believe necroptosis functions as a backup to apoptotic death, while others suggest it is important in generating the antiviral response based on the critical role of Rip3 in promoting inflammation^37^. A specific example of the latter was shown in an influenza infection model, as bone marrow-derived macrophages lacking Rip3 generate a less robust interferon-β response after infection, which is critical in promoting antiviral immunity^38,39^. On the other hand, there is considerable evidence that an abundance of necroptosis perpetuates pathogenic inflammation and drives tissue injury^40^. Rip3 deletion rescues the pathogenesis of several animal models of inflammatory disease, including pancreatitis^28,41^, atherosclerosis^42^, Gaucher’s disease^43^, and morbidity after systemic TNF administration^44,45^, suggesting that necroptosis induces tissue injury by augmenting inflammation after cell death. Fatal cases of SARS-CoV infection similarly show significant lung damage in response to inflammation, which may very well be driven by necroptosis.

The SARS-CoV may have evolved to target Rip3 and the necroptotic pathway for a variety of reasons. First, a late interferon response is critical in the pathogenesis of SARS-CoV^7^, and interferons are produced primarily by immune cells which express high levels of Rip3. SARS 3a-induced deletion of Rip3-expressing cells may suppress the interferon response, allowing better viral survival early in the infection. Second, while necrotic death of IMMs may suppress the interferon response, necroptosis more generally is an inflammatory process. Inflammatory cell death, especially in the lungs, may contribute to the severe lung damage pathophysiologically associated with clinical development of a cough. As SARS-CoV is transmitted primarily through respiratory droplets, propagation of the general inflammatory response via necroptosis may promote virus transmissibility. Finally, necrotic cell death through the generation of pores may perpetuate viral release, as previously suggested^24^.

SARS 3a reduced Rip3 activation and MLKL phosphorylation in our study, suggesting it suppresses the Rip1–Rip3–MLKL axis. While it would be reasonable to expect a reduction in necrotic cell death following Rip3 and SARS 3a co-expression, our cell death assays indicated the opposite. This phenomenon is mechanistically explained by the observation that Rip3 targets SARS 3a to induce its oligomerization, which facilitates SARS 3a membrane insertion and ion channel functionality^24^. We would briefly like to point out that it is possible the oligomers detected in this study are in fact single or multiple SARS 3a proteins in complex with another unknown protein, though it is clear that this complex is needed for cell death. Taken together, our data show that SARS 3a may act as an alternative death effector protein downstream of the Rip3 to induce necrotic death, in effect hijacking the cells necroptotic machinery to promote virus release and inflammatory cell death. As SARS-CoV infection in monocytes and macrophages is typically abortive^46,47^, these data provide an alternative mechanism for host cell lysis, virus release, and cell death.

Despite evidence showing direct interaction between SARS 3a and Rip3, the detailed mechanism behind Rip3′s facilitation of SARS 3a oligomerization is still not clear. Our data sufficiently prove that both wild type and kinase dead Rip3 promotes SARS 3a oligomerization, and that this interaction occurs via the N-terminal Rip3 kinase domain. Rip3 may have a scaffolding role in supporting the formation of SARS 3a oligomers. Typically, the Rip3 kinase dead form (or use of kinase inhibitors) triggers apoptosis due to the RHIM motif-dependent formation of the Rip1/FADD/cFLIP_L_/caspase 8 complex^48^. Given that our results show kinase-independent necrotic death, it suggests the possibility of (exogenous or endogenous) necrotic death effectors downstream of Rip3 that are independent of its kinase activity.

Accumulating evidence shows the importance of IMMs in inducing the aberrant inflammatory state responsible for immunopathologic injury in fatal SARS cases. Infiltrating monocytes (Ly6C^hi^Cd11b^+^CCR2^+^) in SARS-CoV-infected mice have high levels of inflammatory markers (IL-1β, TNF-α, IL-6, and iNOS)^7^. IMMs typically have a proclivity for proinflammatory M1 macrophage differentiation and generate a robust cytokine response after exposure to infectious agents^49,50^. Thus, our study keyed in on SARS 3a to gain molecular level insights into the host–pathogen interactions governing the proinflammatory programming of IMMs. Our data show that SARS 3a activates TFEB. The activation of TFEB may be significant as TFEB directly transcriptionally activates proinflammatory cytokines in macrophages (including IL-1β, IL-2, and IL-27) and promotes a macrophage proinflammatory state^51,52^. TFEB has also been implicated in the migration of macrophages in response to CCL2. CCL2 is a ligand for CCR2, which is expressed highly on SARS-CoV-infected IMMs^51^. Thus, activation of TFEB may contribute to the hypermigration and the inflammatory state of SARS-CoV-infected IMMs.

Finally, our data show that SARS 3a directly initiates the inflammatory cascade by activating the NLRP3 inflammasome and contributing to pyroptotic death. The NLRP3 inflammasome is activated by K^+^ efflux, which causes the kinase NEK7 to translocate to the mitochondrial membrane allowing NLRP3 inflammasome assembly^53^. SARS 3a oligomers likely activate the NLRP3 inflammasome via K^+^ efflux, while SARS 3a monomers directly target and activate caspase-1 to cleave IL-1β. The ability of SARS 3a to generate IL-1β is significant, as IL-1β is an upstream initiator of the inflammatory cascade. It will be of interest to see whether SARS 3a monomers target and activate other caspases, and to determine the contribution of the proinflammatory pyroptotic cell death to SARS pathogenesis. More detailed experiments looking at the cleavage of the gasdermin D protein will be needed to get a better mechanistic understanding of the contribution of pyroptosis to host cell death, but these are quite relevant as both lung IMMs and lung epithelial cells express inflammasome components^54^.

Live virus deletion studies have already shown the importance of ORF-3a in SARS-CoV-induced cell death, intracellular vesicle formation, and SARS-CoV virulence in mouse models^25,26^. Though previous studies have shown that overexpression of SARS 3a alone can recapitulate these phenotypes, the overexpression nature of this study is a limitation that will require further live virus evaluation of the necroptotic pathway for validation. In summary, our data show that a single SARS accessory protein initiates multiple forms of cell death. More significantly, our data strongly link SARS 3a and the SARS-CoV to Rip3, a necroptotic protein whose inhibition has been shown to ameliorate a variety of inflammatory diseases causing tissue damage. This study supports additional research into the contributions of Rip3 and the necroptotic pathway to the pathogenesis of SARS-CoV, and may lay the foundation for a new targeted therapy for SARS or MERS patients.

## Materials and methods

### Reagents and Abs

Cell death was assayed using CellTiter-Glo^®^ Luminescent Cell Viability Assay (Promega, G7572), CytoTox-Fluor™ Cytotoxicity Assay (Promega, G9260), and 7-AAD (ThermoFisher, 00-6993-50) following the manufacturers protocol. E64D (Sigma, E8640) was used at a concentration of 10 μg/ml and Pepstatin A (Sigma, P5318) at 10 μg/ml overnight. Necrosulfonamide (NSA) (Cellagen Technology, C6327) was used at a concentration of 2 μM, Z-VAD (R&D Systems, FMK001) at 20 μΜ, TNF-α at 20 ng/ml (Peprotech, 300-01A), and the Smac-mimetic BV6 (ApeX Bio, B4653) at 10 nM overnight. 5-AD (Sigma, A3656) was used for 4 days at 2 μM. DQ-BSA Red (ThermoFisher, D12051) was used at a concentration of 4 μg/ml following the manufacturer’s protocol, and Cyclosporin A (R&D Systems, 1101) at 10 μM for 18 h.

The following primary Abs were used for western blotting: Anti-Myc Epitope Tag Rabbit (Rockland, 600-401-381, 1:2500), Anti-Flag Epitope Tag Rabbit (Rockland, 600-401-383, 1:5000), Anti-HA Tag Rabbit (Rockland, 600-401-384, 1:5000), Anti-MLKL(D2I6N) Rabbit (Cell Signaling, 1:1000, 14993), Anti-Rip3 Rabbit (E1Z1D) (Cell Signaling, 1:1000, 13526), Anti-MLKL (phospho S358) (Abcam, 1:1000, EPR9514), Anti-Rip3 (phospho S227) (Abcam, 1:1000, EPR9627), Anti-LAMP-1 (H4A3) (Santa Cruz, 1:1000, sc-20011), Anti-Flag monoclonal M2 Mouse (Sigma, 1:500, F1804), Anti-SQSTM1 (P-15) (Santa Cruz, 1:1000, sc-10117), Anti-Myc Mouse (9E10) (Santa Cruz, 1:500, sc-40), Anti-c-Myc Tag (9E10) Affinity Gel (Biolegend, 658502), Anti-TFEB (Bethyl Laboratories, 1:2000, A303-672A), Anti-ASC (2EI-7) (Millipore, 1:500, 04-147), Anti-IL-1β (3A6) (Cell Signaling, 1:1000, 12242), Anti-NLRP3 (D2P5E) (Cell Signaling, 1:1000, 13158), Anti-Caspase-1 (Cell Signaling, 1:1000, 2225).

### Cells and plasmids

HeLa, HEK 293T, A549, and Thp-1 cells were obtained from the American Type Culture Collection. All cells were maintained in Dulbecco's modified Eagle's medium supplemented with 10% fetal bovine serum (Invitrogen), and Thp-1 cells were differentiated with 10 ng/ml PMA overnight prior to use. The vector encoding the SARS-CoV genome was a gift from Marco Marra, and ORF-3a was cloned by PCR and placed into the pEGFP-N1 vector (Addgene). To make the SARS 3a-Flag and SARS 3a-HA constructs, the eGFP was replaced with a 3x Flag tag or HA tag, respectively. Lamp1-mCherry plasmid and LAMP1-CFP plasmids were obtained from the Jenifer Lippincott-Shwartz lab; PPP3CB siRNA was bought from Santa Cruz (SC-39195); the NLRP3 flag plasmid was obtained from Gabriel Nunez’s Lab at the University of Michigan Med School; Caspase-1 flag plasmid, IL-1β-flag plasmid, and ASC-flag plasmids were from the lab of Katherine A Fitzgerald at the University of Massachusetts Medical School; TFEB-GFP was bought from Addgene (38119), and the TFEB-mCherry plasmid was made by moving the TFEB cDNA into the mCherry N1 vector (ClonTech); NEK7 shRNA was made using the targeting sequence: 5′-C​C​G​G​T​G​G​A​G​T​G​C​C​G​G​T​A​G​C​G​T​T​A​A​A​C​T​C​G​A​G​T​T​T​A​A​C​G​C​T​A​C​C​G​G​C​A​C​T​C​C​A​T​T​T​T​T​G​-3′ and inserting into the pSIREN vector (ClonTech); pRip3-mCherry(61386) and ptf-Galectin3 (64149) were purchased from Addgene, and mCherry-Galectin3 was made by removing the GFP from ptf-Galectin3. Rip3-Myc plasmid was a gift of Dr. Zheng-Gang Liu (National Institutes of Health, MD, USA), and truncated constructs were made for the N-terminal (1–326) and C-terminal (327–518). The control vector used was pCR3.1 from Invitrogen. All plasmids were transiently transfected into the cells using X-treme GENE HP DNA Transfection Reagant (Roche, 6366236001) following the manufacturer’s protocol. Cells were transfected overnight (16 h) unless otherwise indicated.

### Immunoblot analysis and immunoprecipitation

For standard immunoblotting, the cells were lysed in Buffer A containing 20 mM HEPES (pH 7.4), 50 mM β-glycerophosphate, 1% (v/v) Triton X-100, 2 mM EGTA, and 10% (v/v) glycerol with a cOmplete protease inhibitor cocktail (Sigma) and PhosStop (Sigma) phosphatase inhibitor tablets. For nuclear fractionation, the cells were lysed with Buffer B, which is the buffer A containing 0.5% (v/v) NP40 instead of Triton X-100 for the cytosolic fraction and with the addition of 0.5% (v/v) sodium dodecyl sulfate (SDS) for the nuclear fraction. For the SARS 3a oligomerization experiments, cells were lysed with Buffer B. The lysates were separated by SDS-polyacrylamide gel electrophoresis (PAGE) on either 4–20% or 8–16% Tris-Glycine gels (Invitrogen) and transferred to a nitrocellulose membrane by the iBlot Gel Transfer System (Invitrogen) using program P2. The membrane was incubated with 5% nonfat milk w/v in TBST buffer (25 mM Tris-HCl, 150 mM NaCl, 0.1% Tween 20) for 1 h, and incubated with the primary Ab in TBST buffer with 2.5% nonfat milk or 5% bovine serum albumin w/v on a shaker overnight at 4 °C. The appropriate secondary Abs conjugated to HRP were used to detect the protein of interest via ECL. For immunoprecipitation experiments, cells were lysed with Buffer C which is Buffer A plus 0.5% (w/v) CHAPS. To immunoprecipitate Myc-Rip3, the lysate was incubated 2 h at 4 °C with the Anti-c-Myc Tag (9E10) Affinity Gel after pre-cleaning with protein G mouse IgG for 30 min. The immunoprecipitates were collected and washed eight times with lysis buffer, separated by SDS-PAGE, and analyzed by immunoblotting. Membranes were stripped using Restore™ Plus Western Blot Stripping Buffer (Thermo Scientific) following the manufacturer’s protocol, re-blocked, and reblotted. Blots were scanned and imported into Photoshop as unmodified tagged image file format. Quantification of band intensity was performed using standard methods on Image J; bands were normalized to their respective loading control and presented as fold change of the control sample.

### Cross-linking

Cells were treated with 500 μl 1.5% formaldehyde solution for 7 min at RT and then pelleted at 1800xg for 3 min at RT, resulting in 10 min of exposure to formaldehyde. The cross-linking reaction was quenched by treating the cells with 0.5 ml ice-cold 1 M glycine in phospate-buffered saline twice, and cells were then lysed in 250 μl RIPA buffer (50 mM Tris-HCl, pH 8.0, 150 mM sodium chloride, 1% NP40, 0.5% sodium deoxycholate, 0.1% SDS, 1 mM EDTA, protease inhibitors) for 60 min on ice. After 30 min, cell lysates underwent 20 strokes using an injection syringe. Lysates were spun for 15 min at 3000 rpm and 4 °C to remove insoluble debris. The supernatant was either used directly or stored at −80 °C. Lysates were separated by SDS-PAGE and analyzed by immunoblotting.

### Confocal microscopy

Transfected cells were seeded in glass-bottom 14 mm microwell dishes (MatTek) and analyzed using confocal microscopy. Live-cell imaging was performed using a Leica DMi8 inverted 5 channel confocal microscope equipped with ultra-sensitive hybrid detectors (Leica Microsystems) and an Environmental Chamber (NIH Division of Scientific Equipment and Instrumentation Services) to maintain 37 °C and 5% CO_2_. The following laser lines were used at minimal laser power (0.2–1%): diode for 405 nm, Argon for 458 and 488 nm, and HeNe lasers for 594 and 633 nm excitation wavelengths. To observe Rip3-dependent cell death in SARS 3a-expressing cells, SARS 3a-GFP was transfected on day 0 and Rip3-mCherry transfected on day 1. Recordings were done overnight after day 1 transfection, and the first time point with Rip3-mCherry expression was considered time 0. For time-lapse analysis of protein expression in the cells, Z-stacks of cellular monolayers (*Z* = 10–15 µm) were collected over time (1 to 12 h). Post-acquisition image processing and analysis of protein colocalization was performed using Huygens (Scientific Volume Imaging) and Imaris (Bitplane) software. For quantification of the BSA Red substrate, circular regions of interest were defined and analyzed for intensity using LASAF software (Leica) in transiently transfected SARS 3a-GFP cells and untransfected cells from the same dish; the same analysis was performed on cells transiently transfected with GFP-vector or untransfected cells from the same dish as a control. Data are normalized to the GFP-negative cells from the control dish and presented as Normalized Intensity. For galectin-3 spot analysis, the spot function of Imaris Cell was used to identify and quantify cytoplasmic spots greater than 0.75 μm in diameter following 3D reconstruction of *z*-stacks. A minimum of 15 cells are included for all imaging quantification.

### qRT-PCR

Total RNA extraction was done using Trizol (ThermoFisher scientific, 15596026) following the manufacturers protocol. Two micrograms of extracted RNA was used the same day for cDNA synthesis using the Omniscript RT Kit (Qiagen, 205113). Quantitative real-time PCR (qRT-PCR) was performed in 10 ul reactions containing 2× SYBR Green Master Mix, diluted cDNA, and PCR primers. The primers used for qRT-PCR were as follows:

GAPDH: F′: TGCACCACCAACTGCTTAGC, R′: GGCATGGACTGTGGTCATGAG;

TFEB: F′: CCAGAAGCGAGAGCTCACAGAT, R′: TGTGATTGTCTTTCTTCTGCCG;

SQSTM1: F′: GCACCCCAATGTGATCTGC, R′: CGCTACACAAGTCGTAGTCTGG;

LAMP1: F′: ACGTTACAGCGTCCAGCTCAT, R′: TCTTTGGAGCTCGCATTGG;

ATP6V1H: F′: GGAAGTGTCAGATGATCCCCA, R′: CCGTTTGCCTCGTGGATAAT. Reaction mixtures were incubated at 95 °C for 10 min, followed by 40 cycles of 95 °C for 10 s and 60 °C for 1 min, followed by a melting curve stage. A threshold cycle (Ct) was observed in exponential phases of the amplification, and quantification of the relative expression levels was determined by the 2^−△△Ct^ method.

### Cell death by protease release and ATP depletion

HEK 293T cells or HeLa Cells were seeded into an opaque-wall 96-well plate (Plate A) and transiently transfected; inhibitors were added 3 h after transfection. After 24 h, 50 μl of supernatant from each well was transferred into a new opaque-wall 96-well plate (Plate B). Fifty microliters of CytoTox-Fluor™ Cytotoxicity Assay reagent was added to test leakage after cell membrane damage. After incubation at 37 ℃ for 1 h, plates were read on a FlexStation 3 at 485nmEx/520nmEm. Concurrently, plate A was equilibrated at room temperature for 30 min, followed by the addition of 50 μl CellTiter-Glo® Luminescent Cell Viability Assay reagent into each well of plate A to test the ATP depletion of live cells. After mixing contents for 2 min on an orbital shaker to induce cell lysis and a 10-min incubation to stabilize luminescent signal, the luminescence was recorded using the FlexStation 3. Data were normalized to the vector control and reported as percent change compared to the control.

### Statistical analysis

One-way ANOVA followed by post hoc Tukey’s HSD test was used to test for statistical differences between all groups. All statistical analysis was done on GraphPad Prism 7.01 and *p* < 0.05 was considered significant. Experiments were repeated a minimum of three times.

## Electronic supplementary material

Figure 1S

Figure 2S

Figure 3S

Video 1 - SARS 3a and RIP3

Video 2 - TFEB-mCherry and SARS 3a-GFP

Supplementary Figure Legends
