# Supplementary figures and images for: SARS-Coronavirus Open Reading Frame-3a drives multimodal necrotic cell death
Source: Cell Death Dis. 2018 Sep 5;9(9):904. doi: 10.1038/s41419-018-0917-y (PMC6125346; doi:10.1038/s41419-018-0917-y)

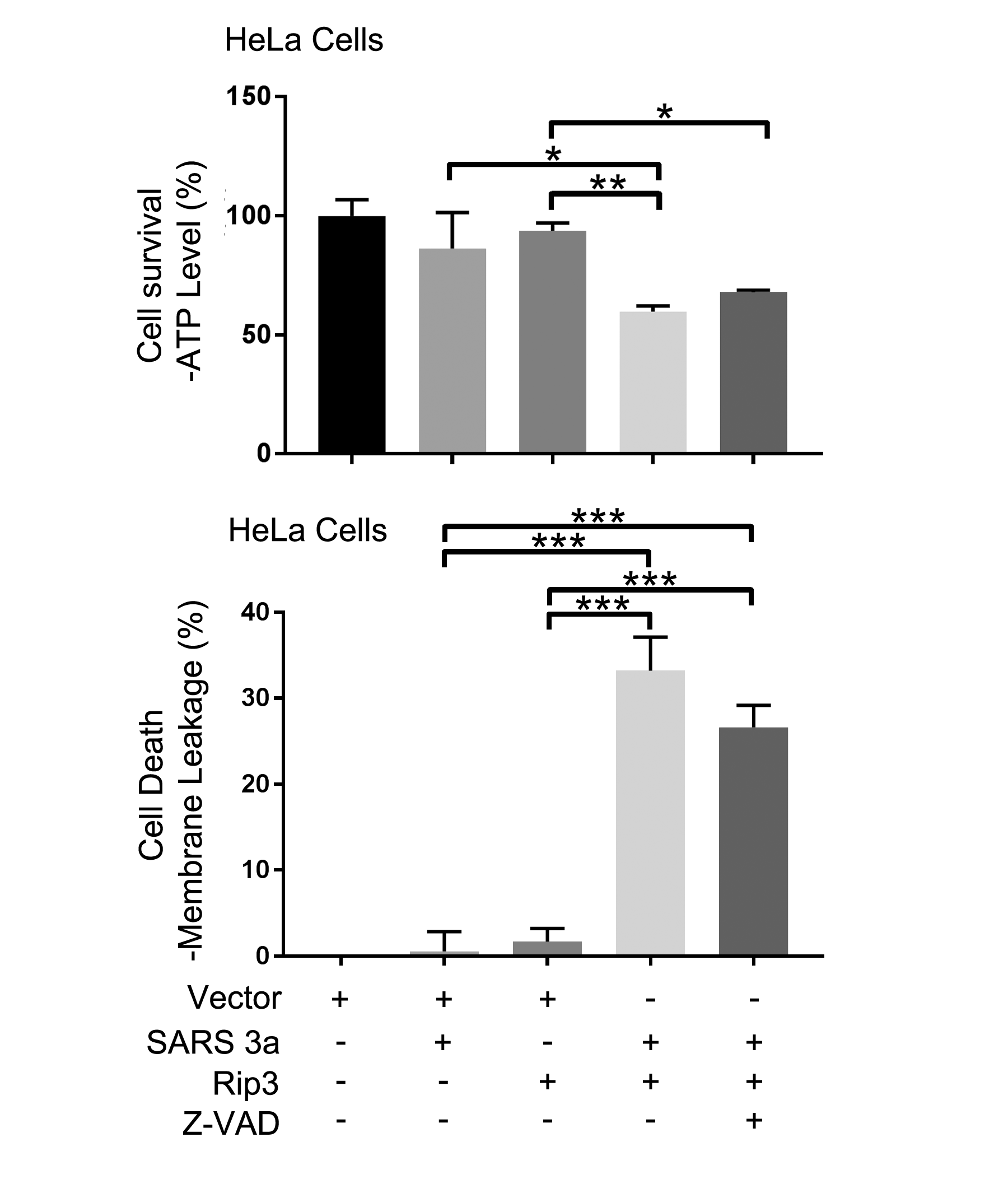

Supplement: Supplementary file 1 — Figure 1S [file 41419_2018_917_MOESM1_ESM.tif]

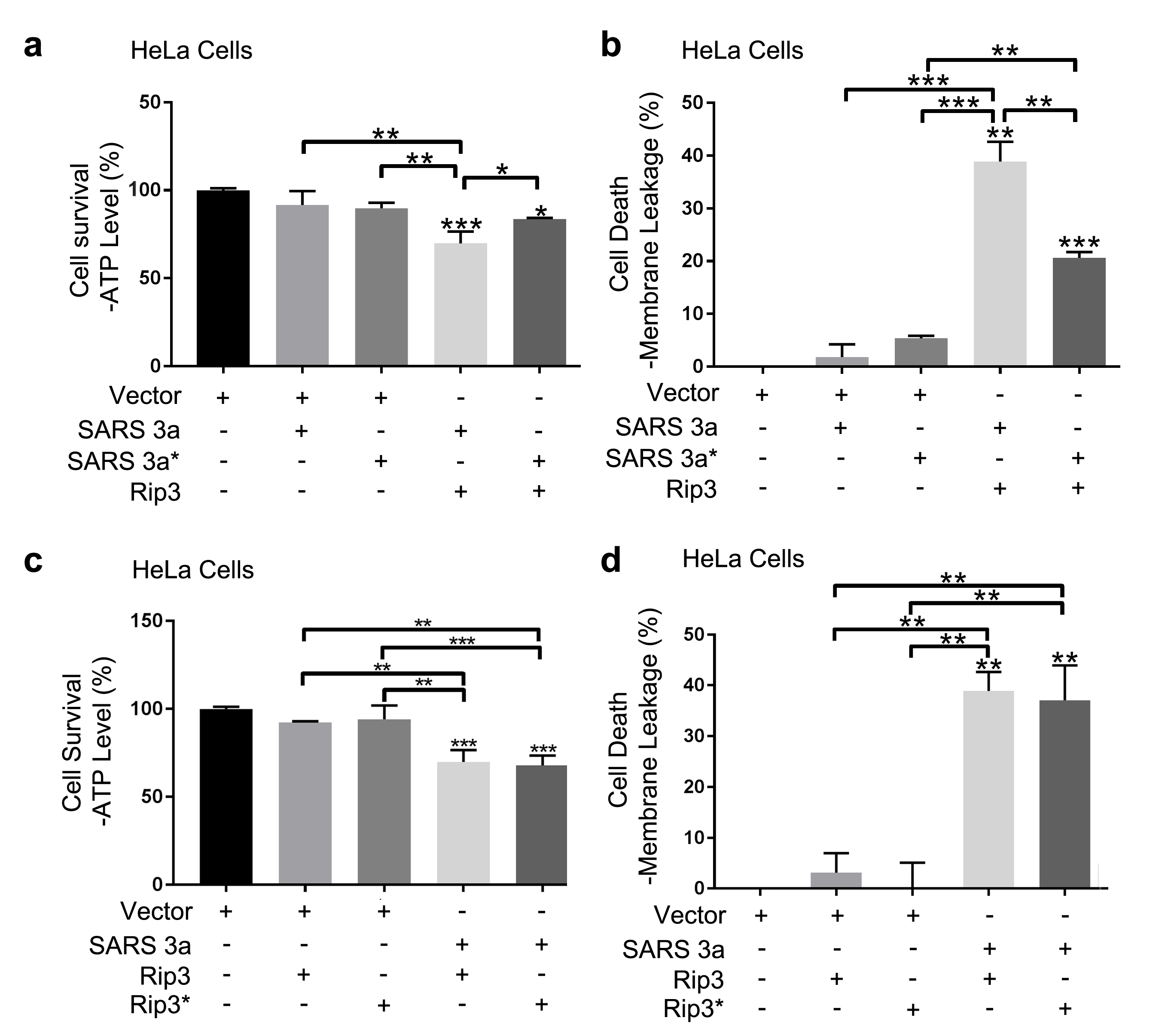

Supplement: Supplementary file 2 — Figure 2S [file 41419_2018_917_MOESM2_ESM.tif]

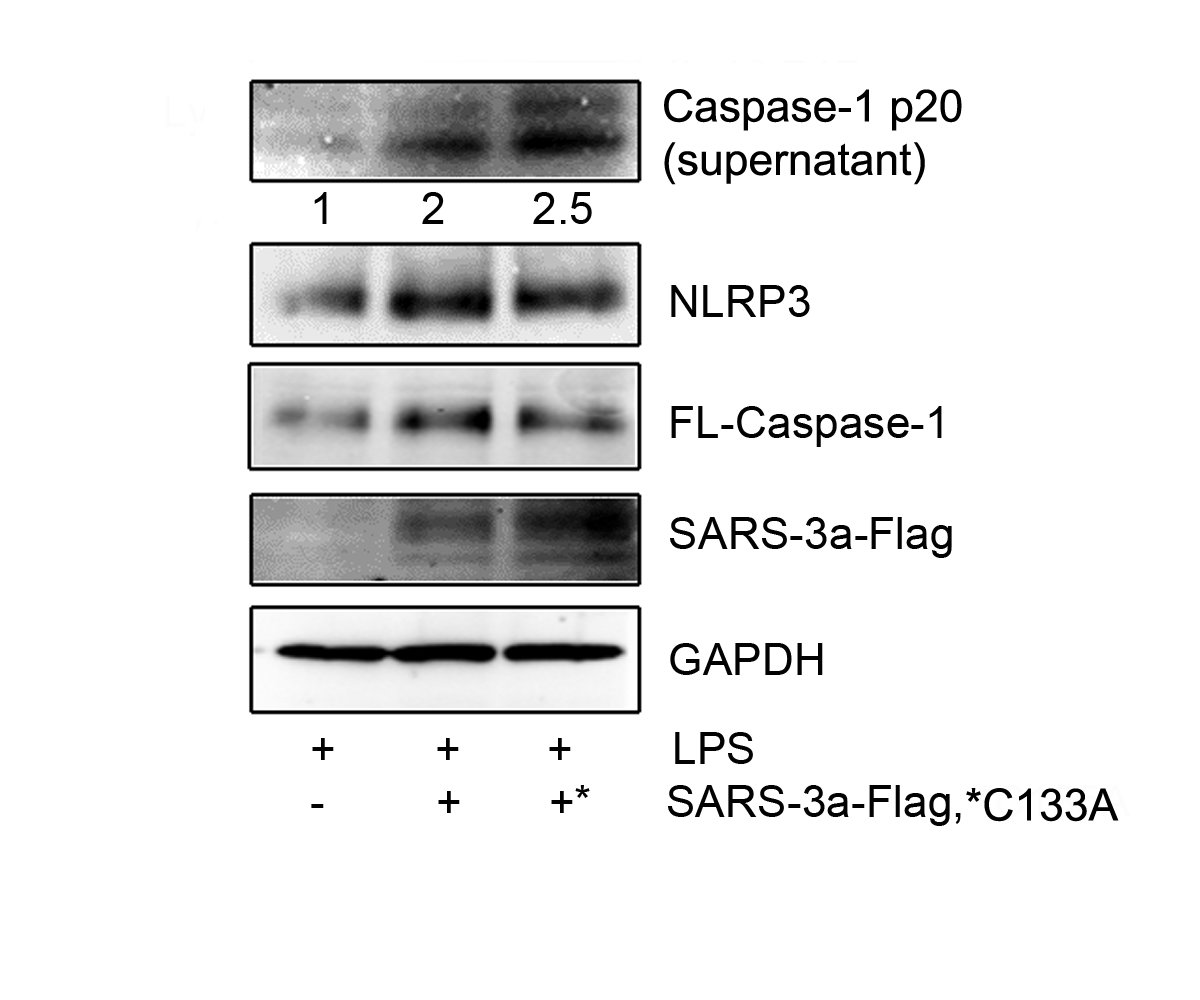

Supplement: Supplementary file 3 — Figure 3S [file 41419_2018_917_MOESM3_ESM.tif]
