# Supplementary material for: SARS-Coronavirus Open Reading Frame-3a drives multimodal necrotic cell death
Source: Cell Death Dis. 2018 Sep 5;9(9):904. doi: 10.1038/s41419-018-0917-y (PMC6125346; doi:10.1038/s41419-018-0917-y)
Supplement: Supplementary file 6 — Supplementary Figure Legends [file 41419_2018_917_MOESM6_ESM.docx]

**Supplementary Figure Legends**

**Figure S1.** SARS 3a drives RIP3 dependent necrotic cell death. Cell death assays (ATP level and membrane leakage assays) in HeLa cells after co-transfection of SARS 3a and Rip3, with and without caspase inhibitor. Cell death data are the average and SEM of n = 3 independent experiments in triplicate. (*p < 0.05; **p < 0.001, ***p < 0.0001, ordinary one-way ANOVA with post hoc Tukey’s HSD).

**Figure S2.** SARS 3a induced cell death is SARS 3a oligomerization dependent and Rip3 kinase independent. (**a,b**) Cell death assays (ATP level and membrane leakage assays) in HeLa cells following cotransfection of RIP3 and either SARS 3a WT or SARS 3a C133A (SARS 3a*) (**c,d**) Cell death assays after cotransfection of SARS 3a and either RIP3 WT or RIP3 KD (kinase dead). Cell death data are the average and SEM of n = 3 independent experiments in triplicate. (*p < 0.05; **p < 0.001, ***p < 0.0001, ordinary one-way ANOVA with post hoc Tukey’s HSD).

**Figure S3.** SARS 3a induces caspase-1 cleavage in macrophages. Immunoblot analysis of inflammasome components in the lysate and cleaved-caspase 1 (p20) in the supernatant after SARS 3a transfection into PMA-differentiated Thp-1 cells.

**Video 1.** SARS 3a and Rip3 dynamically drive cell death. Time lapse confocal microscopy of SARS 3a-GFP and Rip3-mCherry dynamically showing cell death after Rip3-mCherry expression in SARS 3a expressing cells.

**Video 2.** SARS 3a drives TFEB nuclear translocation. Time lapse confocal microscopy showing the translocation of mCherry-TFEB upon expression of SARS 3a-GFP.
